# Assessing phonological short-term memory in Greek: Reliability and validity of a non-word repetition test

**DOI:** 10.3389/fpsyg.2022.904268

**Published:** 2023-02-21

**Authors:** Ioanna Talli, Panagiota Kotsoni, Stavroula Stavrakaki, Liliane Sprenger-Charolles

**Affiliations:** ^1^Department of Italian Language and Literature, Aristotle University of Thessaloniki, Thessaloniki, Greece; ^2^Laboratoire de Psychologie Cognitive (CNRS UMR 7290), Aix-Marseille University, CNRS, Marseille, France

**Keywords:** non-word repetition, validity, reliability, reading fluency, phonological short-term memory (STM)

## Abstract

This study explores the reliability and validity of a NWR task in a large cohort of 387 TD Greek-speaking children aged 7–13 years attending elementary (Grades 2–6) and secondary school (Grade 1), divided into six age groups. Further, the relationship between NWR and reading fluency skills as well as the predictive value of the NWR on reading fluency skills in TD children are examined. To investigate the external reliability of the NWR task, test-retest reliability was performed, and excellent test-retest reliability was found. Internal reliability was explored with Cronbach’s alpha coefficient and good reliability was found. To explore convergent validity, correlation analysis between NWR and reading fluency was conducted and significant and strong correlations were found for all age groups excepted 2 (ages 9-10 and 12-13). To examine predictive validity, regression analysis was conducted between these two variables and showed that performance on NWR contributed significantly to reading fluency skills, suggesting that NWR skills are a good predictor of reading skills. Finally, it was explored whether the relevant scores increase as a function of age and found significant differences between groups that differed in 2 years or more, while this difference was no longer significant after 10 years. This finding suggests that phonological STM increases in capacity along with age, but only until the age of 10, where it seems to reach a ceiling. In addition, linear regression analysis showed that age contributed significantly to performance on NWR test. To sum up, the present study provides normative data of a NWR test for a wide age range, which does not exist in the Greek language (particularly for ages over 9 years) and it can be concluded that the present NWR test can be successfully used as a reliable and valid measure of phonological STM in the age range that was examined in this study.

## Introduction

The non-word repetition (NWR) task has been widely studied in the fields of both typical and atypical language development, including language and reading disorders (for review see Coady and Evans, 2008). The task of NWR includes listening to and repeating novel phonetic sequences (non-words), which are built upon the rules of a language’s phonotactic structure. NWR stimuli have sometimes included real words, given language-specific constraints on consonants, vowels, and syllable structure (Ebert et al., 2008).

The nature of the NWR task is a matter of high controversy in the relevant literature and as a result, various hypotheses have been developed as to which skills and processes are involved while performing a NWR task, with research suggesting phonological short-term memory (STM; Gathercole and Baddeley, 1990), phonological working memory (Montgomery, 1995; Bishop et al., 1996; Dollaghan and Campbell, 1998; Botting and Conti-Ramsden, 2001), phonological encoding (Kamhi and Catts, 1986), phonological awareness or sensitivity (Metsala, 1999), or a general phonological processing ability (Bowey, 2001).

### Non-word repetition and linguistic abilities

It has been suggested that multiple processes are involved in NWR, which concern mainly linguistic abilities, such as (in order of appearance) encoding, temporary storage, retrieval, and articulation (Snowling et al., 1991; Gathercole et al., 1994; Edwards and Lahey, 1998; Briscoe et al., 2001). More specifically, Coady and Evans (2008) describe this process as follows: “*The repetition of non-word stimuli involves speech perception, phonological encoding (or segmenting the acoustic signal into speech units that can be stored in memory), speech motor planning (formulating a motor plan of relevant speech units assembly), and articulation.”* Moreover, a representation of specific speech units and memory skills are required, so that the novel phonological string can be stored and operated. If any of these component skills that are involved is affected in any way, then the child’s ability to repeat a novel word would be affected, too.

More recently, researchers have suggested that NRW is actually a reflection of the child’s language exposure, bringing out a major linguistic component (e.g., Jones, 2016; Jones and Macken, 2018; Szewczyk et al., 2018). There are authors that propose that NWR skills rely considerably on lexical knowledge (Roodenrys and Hinton, 2002; Gathercole, 2006) and that wordlikeness (the similarity between an existing word and a pseudoword) is a factor that needs to be considered in NWR tasks (Gathercole, 1995; Munson et al., 2005). Others suggest that NWR is supported by representations not only at the lexical level but also at the sublexical level (Jones et al., 2014; Jones, 2016), indicating that children store sublexical representations of various lengths (i.e., sequences of phonemes) and that the greater the exposure to a certain language, the longer the sequences of phonemes stored. In this respect, some factors that need to be considered are phonotactic frequency (Gathercole et al., 1999; Edwards et al., 2004; Metsala and Chisholm, 2010), prosody (Roy and Chiat, 2004), and syllable complexity (Marshall and van der Lely, 2009).

### Linguistic properties in non-word repetition tasks: studies with bilingual and clinical populations

NWR has been widely used in bilingual populations in order for the effects of language knowledge on it to be examined (Chiat, 2015, for a review), with findings to remain contradictory so far, while, NWR skills in clinical populations seem to be impaired as well.

According to research findings, it has been indicated, on the one hand, that language experience has little or no effect on NWR performance (e.g., Lee et al., 2013; Thordardottir and Brandeker, 2013), as no difference in the performance in the NWR task between bilingual and monolingual preschool age children was found, while on the other hand, that language experience has a negative effect on NWR performance (Lee and Gorman, 2012; Sharp and Gathercole, 2013) and that bilingual children perform worse in NWR than their monolingual peers (Kohnert et al., 2006; Windsor et al., 2010; Engel, de Abreu, 2011).

Additionally, NWR task is considered to be a marker of Developmental Language Disorder (DLD) (for a meta-analysis see: Schwob et al., 2021) in studies conducted in English (e.g., Bishop et al., 1996; Weismer et al., 2000; Conti-Ramsden et al., 2001), as well as in other languages (e.g., Arabic: Taha et al., 2021; French: Thordardottir and Reid, 2022; Italian: Bortolini et al., 2006; Spanish and Portuguese: Girbau, 2016; Ahufinger et al., 2021; Vietnamese: Pham and Ebert, 2020).

Children with DLD have more difficulty with some linguistic factors such as syllable complexity (for instance, the presence of consonantal clusters: Archibald and Gathercole, 2006; Jones et al., 2010) or low phonotactic probability (also known as phonotactic frequency, i.e., the frequency of the sequences of phonemes of a word or a non-word) (Munson et al., 2005), but findings are not stable across studies (Jones et al., 2010). Moreover, as far as non-word length is concerned, children with DLD have more difficulty with longer non-words (Graf Estes et al., 2007), but this varies according to the length range of the non-words compared. There are languages that contain more monosyllable or disyllable words (e.g., English), while other languages contain more multisyllable words (e.g., Italian, Spanish, German, French, etc.). As a consequence, children’s experience of word length is different from language to language, which might affect the ability to repeat longer non-words in one language but not in another (Summers et al., 2010; Dispaldro et al., 2013).

Further, NWR skills are also associated with reading impairment (e.g., Melby-Lervåg and Lervåg, 2012; Ehrhorn et al., 2021). Deficits in NWR are found in children with reading impairment with or without language impairment and they are more severe in those with both language and reading impairment (Catts et al., 2005), but not in those with language impairment with no reading impairment (Baird et al., 2011). However, two other studies conducted in Greek and in French that have compared children with DLD and children with reading impairment with TD same-age and same reading level controls have shown that both had deficits in NWR skills, being more severe in children with DLD (Talli et al., 2015, 2016).

### Non-word repetition and reading fluency

Strong heritable influence on reading acquisition has been found for children with poor NWR skills (Bishop et al., 2004) and performance on NWR has been associated with reading skills (Conti-Ramsden and Durkin, 2007). For example, Maridaki-Kassotaki (2002) studied the relationship between phonological STM and reading ability in TD Greek-speaking children between 6 and 9 years and found a significant and strong relationship. Respectively, poor NWR performance has been associated with poor reading skills (decoding and reading fluency) in children with dyslexia (Talli et al., 2015, 2016). Children with reading impairment have poor phonological STM skills (Menghini et al., 2011; Schuchardt et al., 2013; Fischbach et al., 2014). These phonological STM deficits are thought to impede the acquisition of letter-sound correspondences that are necessary for acquiring decoding skills (Brady, 1986; Rack et al., 1992).

NWR has been successfully used as an early predictor and an accurate identifier of children at risk for reading disorders. More specifically, Catts et al. (2015) administered a battery of tests, including an NWR task, to 366 children attending kindergarten and assessed a subset of them (263 children) again at the end of first Grade. They found that NWR test (along with measures of letter naming fluency, phonological awareness, and rapid naming) could predict reading fluency skills and could identify successfully good and poor readers. Differences in performance in NWR between good and poor readers can be explained by the efficiency of underlying phonological processes, which are less accurate in poor readers (Rapala and Brady, 1990). Except for phonological STM, phonological awareness is also a component of these underlying phonological processes that are related to NWR skills. More specifically, NWR skills have been shown to predict phonological awareness skills (Erskine et al., 2020). Elhassan et al. (2017) explored whether phonological awareness correlates with reading fluency and whether it can predict reading fluency skills in fluent, moderate fluent, and dysfluent readers aged 9–12 years. They found that phonological awareness contributed significantly only for dysfluent readers, suggesting that, once automaticity in reading is achieved, phonological awareness skills no longer affect reading skills. All the above-mentioned findings demonstrate the importance of considering phonological skills in good and poor readers.

### Non-word repetition in Greek

As regards the Greek language in NWR tests, research is limited with little attention to be given to the diagnostic value of NWR tasks. More specifically, there are only few studies examining NWR skills of typically developing (TD) children (e.g., Masoura and Gathercole, 1999; Gathercole and Masoura, 2005; Masoura, 2006) and of clinical populations such as DLD and/or SLD in the native language (e.g., Lalioti et al., 2016; Talli et al., 2016; Mengisidou and Marshall, 2019; Talli and Stavrakaki, 2020) and in the second language (Kotsoni, 2021) as compared to TD children, with the studies being conducted mainly by employing non-standardizing measures of NWR tests. Further, as regards the diagnostic value of the NWR test, several studies have shown that TD students’ performance in an NWR task (in Greek) differed significantly from that of students with reading impairment (Talli et al., 2016; Kotsoni, 2021; Masoura et al., 2021) and to students with DLD (Lalioti et al., 2016; Talli et al., 2016; Talli and Stavrakaki, 2020). Additionally, regarding the predictive value of the NWR task in the study, Kotsoni (2021) indicated that the NWR task in Greek significantly predicted TD and reading impaired students’ second language (L2) vocabulary learning (English) in an inclusive environment. Since phonological STM has been repeatedly shown to affect and predict vocabulary development, it has been argued that the ability to repeat a non-word (or a novel word in vocabulary acquisition) considerably depends on phonological STM capacity and that the main function of phonological STM is to support word learning (Gathercole and Baddeley, 1989, 1990; Gathercole et al., 1997; Baddeley et al., 1998). Thus, NWR tasks should be further explored as to its relation to both language learning and especially its role in non-typical language development through standardized NWR tests.

To the best of our knowledge, the only Greek standardized NWR test, which is the subscale of a screening test of reading difficulties, is the one developed by Porpodas (2007) but only assesses children’s NWR capacity aged 5–7 years old. It consists of 24 non-words, 2–5 syllables length. Further, there are also non-standardized NWR tasks in Greek that have been administered to children of 5–7 years (Masoura et al., 2004), 6–9 years (Maridaki-Kassotaki, 2002) and 5.5–9.5 years (Chrysochoou, 2006). The first, based on Children’s Non-word Repetition Test (CNRep; Gathercole et al., 1994), consists of 50 non-words, 2–6 syllables length, 10 for each length. The second, which was also based on CNRep test of Gathercole et al. (1994), consists of 40 non-words, 2–5 syllables, 10 for each length. The third, based on WM Test Battery for Children (WMTB-C; Pickering and Gathercole, 2001), adapted to Greek (by Chrysochoou, 2006), consists of two-syllable non-words, given in lists of one to six non-words. Hence, taking into consideration, the relation of NWR tasks to (a) both typical and non-typical, first and foreign language development and (b) the fact that only one standardized NWR test exists in the Greek language only for children of a limited age range (5–7 years old), it may be argued that there is an urgent need for standardized tests in order to assess children aged 7–9 years old through a valid and reliable NWR test and also to extend the age range in populations over 9 years old as an NWR test for ages over 9 years old is non-existent in the Greek language. Normative data of NWR skills in older children and adolescents can be informative in the assessment and identification of clinical populations, i.e., dyslexia or DLD (Goulandris et al., 2000; Snowling et al., 2000; Botting and Conti-Ramsden, 2001; Ebbels et al., 2012; Melby-Lervåg and Lervåg, 2012; Nielsen et al., 2016; Thordardottir and Reid, 2022).

The aim of our study is to bridge that gap of normative data of NWR tests from Greek-speaking populations, by testing the reliability and the validity of a test of NWR in a large number of children of a wide age range (7–13 years). This task can be used for educational, clinical, as well as research purposes.

The specific research questions that this study addresses are the following:

1. Is this NWR test a reliable measure for TD Greek-speaking children aged 7–13 years?
2. Is this NWR test a valid measure for TD Greek-speaking children aged 7–13 years? What is the relationship between NWR and reading fluency skills in TD children aged 7–13 years? Can this NWR test predict reading fluency skills?
3. What is the relationship between age and performance in NWR test? Is there an escalation in performance as a function of age group? Can age predict performance in NWR test?

Our first research question will be examined by performing test–retest reliability analysis to check for external validity and Cronbach’s alpha coefficient to examine internal reliability of the NWR test. our first research question (reliability), test-retest reliability analysis will be performed to explore external reliability and Cronbach’s alpha coefficient to explore internal reliability. As regards our second research question, correlation analysis between NWR and reading fluency will be conducted to check for convergent validity, as well as regression analysis between these two variables will be conducted to examine predictive validity. Our third research question, concerning the relation between age and NWR skills, will be examined by performing ANOVAs and *post-hoc* tests, as well as linear regression analysis.

Our hypotheses are that the present NWR test will show high test-retest reliability and excellent or at least good internal consistency, suggesting that it is a reliable measure for TD Greek-speaking children aged 7–13 years. We also predict that it is a valid measure for TD children of this age range: significant and strong correlations are expected to exist between NWR and reading fluency skills for all groups (convergent validity). Moreover, we anticipate that NWR performance will be able to predict reading fluency performance (predictive validity), suggesting that the NWR test can screen for children with or without reading impairment. Given the fact that as children grow older, their memory capacity increases (Gathercole, 1998), we predict that NWR performance will escalate as a function of age group, i.e., the older the children, the better the performance.

## Materials and methods

### Participants

The participants of this study were 387 TD children (206 girls/181 boys) aged 7–13 years attending elementary (Grades 2–6) and secondary school (Grade 1) that were examined and divided into six age groups: 7–8, 8–9, 9–10, 10–11, 11–12, and 12–13 years. None of them had a history of speech and language problems, no diagnosis of neurological, motor, or sensory disorder (such as hearing loss), and no additional learning difficulties. All children had normal non-verbal IQ and scored above the 25th percentile in Raven Progressive Matrices, a test of non-verbal IQ (Raven, 2003; Greek standardization; Sideridis et al., 2015). They were considered to be typically developing children by parents, teachers, and foreign language teachers. They were monolingual children and their first language was Greek. They were randomly recruited from ten different schools (four public elementary schools and six private foreign language schools) in seven different cities, towns, and villages (urban, semi-urban and rural areas) in three different prefectures in the region of Northern Greece (Thessaloniki, Chalkidiki, and Pella), while the participants’ families were of diverse socio-economic status. All typical children, aged 7–13 years, from these schools, after non-verbal IQ assessment and parents’ written consent, were included in the sample.

### Materials

#### Non-word repetition task

This test was adapted in Greek from the French test battery EVALEC (Sprenger-Charolles et al., 2005; Greek adaptation: Talli, 2010) and it consisted of 24 three- to six-syllable pre-recorded non-words presented through headphones connected to a computer in order of increasing length (some examples, one for each length: povidu, todokino, tabaritoli, madurlanoti). The children had to repeat each item with no time constraint. The total number of syllables correctly repeated was the accuracy score, calculated in percentages. For the Greek adaptation of the non-word repetition task from the test battery EVALEC we maintained the number of non-words, the number of syllables, as well as the phonotactic structures (the syllabic structures used were CV, CVC, and CCV).

#### Reading fluency test

A reading fluency test was additionally administered (“Giro Giro oli,” adaptation of “Alouette,” Lefavrais, 1967; Talli, 2010; Talli et al., 2015), in which children were asked to read aloud a 271-word text as accurately and rapidly as possible and we calculated a composite score by adding the total number of non-corrected errors and the total number of non-read words to the total reading time (with a limit of 180 s). The higher the score, the worse the performance.

### Procedure

Participants were assessed individually by experienced special education teachers and researchers in one session of 20 min. Assessment took place in a private room in children’s schools. All parents gave written consent for their children to participate in the study.

## Results

### Non-word repetition test as a reliable measure for Greek-speaking typically developing children aged 7–13 years

The first research question regarded the reliability of the NWR test for Greek-speaking TD children aged 7–13 years. A test-retest reliability test was performed, which examines the external reliability of a method or an instrument. The 60 participants (10 of each group) were examined twice with a 3-week time difference (to alleviate between the history effect, on the one side, and the age (month measurement), on the other. Correlation between the first (*M* = 91.39, *SD* = 5.58) and the second measurement (*M* = 91.71, *SD* = 5.38) showed a strong and significant relationship (*r* = 0.913, *p* < 0.001). Consequently, the results of the test-retest reliability correlation showed excellent reliability of the instrument between the two times measurements.

Moreover, to examine the internal reliability of the NWR test, we performed Cronbach’s alpha coefficient analysis to 60 participants (10 for each age group) We calculated for each participant the performance in each of the 108 items of the test, giving score 1 for each correct item and score 0 for each incorrect item. NWR test showed good internal consistency (a = 0.81).

### Relationship between non-word repetition and reading fluency skills in typically developing children 7–13 years

To investigate the validity of the NWR test, convergent and predictive validity were examined.

First, convergent validity was assessed by testing associations between NWR and the reading fluency test (Otto et al., 2011; Cecil et al., 2015; Cavalli et al., 2018) in TD children, by performing Pearson correlation coefficients. First, a correlation was calculated between the two scores in the whole group. The results between NWR (*M* = 91.55, *SD* = 7.08) and the reading fluency test (*M* = 225.45, *SD* = 72.99) indicated a significant and strong correlation *r* = –0.53, *p* < 0.001, confirming, thus, the convergent validity of the NWR test to screen for TD children. Second, correlations were calculated across the six age groups. Significant correlations between the NWR test and the reading fluency test were found for all age groups (*r* = –0.36, –0.37, –0.50, and –0.29, for groups 7–8, 8–9, 10–11, and 11–12, respectively) except for those of 9–10 for which there was a non-significant but weak relationship (*r* = –0.22) and of 12–13 years for which there was a non-significant relationship (*r* = –0.16).

In Table 1, descriptive statistics for age and performance in the NWR and reading fluency task in each of the six age groups are displayed. In Table 2, Pearson Correlations analysis between the NWR test and the reading fluency task for each age group are presented.

**TABLE 1:** Means (SDs) of age (in months) and performance in the NWR task and reading fluency (RF) task in each of the six age groups.

Measure	7–8^a^ (N = 54)	8–9^b^ (N = 64)	9–10^c^ (N = 70)	10–11^d^ (N = 61)	11–12^e^ (N = 75)	12–13^f^ (N = 63)
	M *(SD)*	M *(SD)*	M *(SD)*	*M (SD)*	*M (SD)*	*M (SD)*

1. Age (in months)	88.63 (4.10)	101.81 (4.00)	114.89 (3.26)	126.31 (3.45)	138.19 (3.06)	150.92 (3.72)
2. NWR	86.15 (6.75)	88.54 (8.56)	90.75 (7.26)	92.96 (5.31)	94.32 (5.04)	95.46 (4.50)
3. RF	331.07 (41.43)	285.48 (46.42)	242.87 (52.76)	193.16 (28.18)	172.65 (29.19)	148.68 (21.48)

Significant differences; Age: (1a–1b)***; (1b–1c)***; (1c–1d)***; (1d–1e)***; (1e–1f)***, NWR: (2a–2c)***; (2b–2d)***; (2c–2e)***; RF: (3a–3b)***; (3b–3c)***; (3c–3d)***; (3d–3e)*; and (3e–3f)*. *p < 0.05, **p < 0.01, and ***p < 0.001.

**TABLE 2:** Descriptive Statistics and Pearson Correlations for NWR task with reading fluency (Correlation for children as a whole group: −0.53, *p* < 0.001).

Variable	*N*	*M*	*SD*	RF r
1. NWR 7–8	54	86.15	6.75	–0.36**
2. NWR 8–9	64	88.54	8.56	–0.37**
3. NWR 9–10	70	90.75	7.26	–0.22
4. NWR 10–11	61	92.96	5.31	–0.50**
5. NWR 11–12	75	94.32	5.04	–0.29**
6. NWR 12–13	63	95.46	4.50	–0.16

** *p* <0.01.

Further, to investigate the predictive validity of NWR, a linear regression analysis was performed to check if the NWR test was a significant predictor of the reading fluency test. The regression was statistically significant [*R*^2^ = 0.526, *F*(1, 385) = 147.34, *p* < 0.001]. It was found that the NWR test [B = –5.42, *p* < 0.001) significantly predicted participants’ reading fluency which means that for each unit of the NWR, we expect, on average, the reading fluency score to decrease significantly (which means improved reading fluency) at about 5.42 points.

**FIGURE 1:**
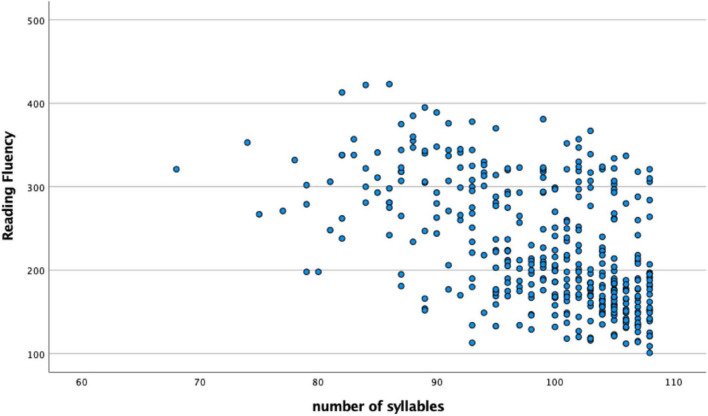
Scatterplot showing the performance of all age groups (387 participants) in NWR test (*Y*-axis: dependent variable) in relation to reading fluency task (*X*-axis: independent variable).

**FIGURE 2:**
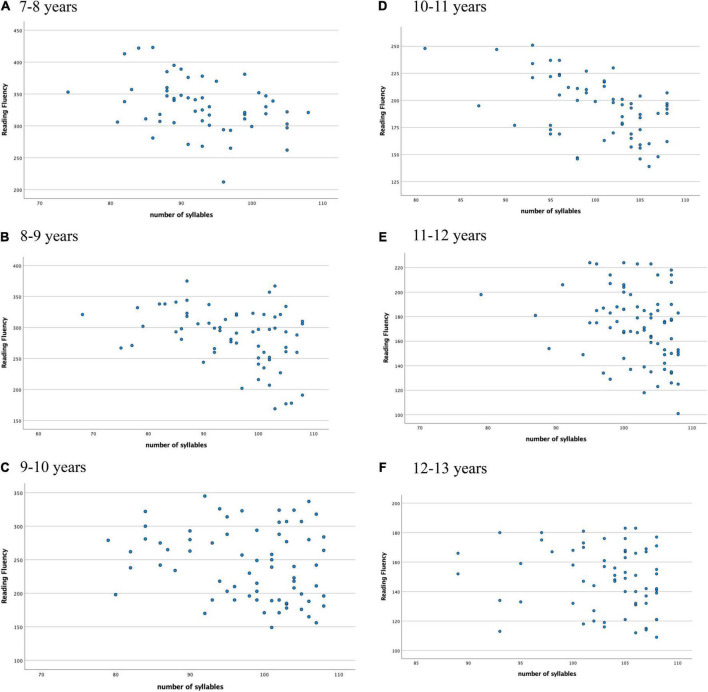
Scatterplots **(A–F)** showing the performance of each age group (7–8, 8–9, 9–10, 10–11, 11–12, 12–13, respectively) in NWR test (*Y*-axis: dependent variable) in relation to reading fluency task (*X*-axis: independent variable): **(A)** 7–8 years, **(B)** 8–9 years, **(C)** 9–10 years, **(D)** 10–11 years, **(E)** 11–12 years, **(F)** 12–13 years.

Finally, Figure 1 (scatterplot) shows the distribution of all groups’ performances in NWR test in relation to reading fluency task, while Figure 2 (scatterplots A–F) shows this distribution for each of the age groups (7–13 years old).

### The relationship between age and performance in non-word repetition test

Our third research question attempted to examine the relationship between age and performance in the NWR test and, more specifically the escalation (classification) of children’s performance as a function of age group and the predictive value of age over the NWR test. In order to investigate these hypotheses, two different statistical tests were performed, a one-way Anova test and a regression analysis.

#### One-way Anova

One-way Anova test indicated that the main effect of the NWR task was significant [*F*(5, 381) = 18.98, *p* < 0.001]. *Post-hoc* tests indicated significant differences between all groups that differed in 2 years or more. This difference was no longer significant after 10 years of age (see Tables 1, 3).

**TABLE 3:** *Post hoc* comparisons (Tukey’s HSD) of NWR in the six groups.

				Tukey’s HSD Comparisons				
Group	*N*	Mean	*SD*	7–8	8–9	9–10	10–11	11–12
7–8	54	86.15	6.75					
8–9	64	88.54	8.56	0.325				
9–10	70	90.75	7.26	0.001	0.341			
10–11	61	92.96	5.31	<0.001	0.002	0.360		
11–12	75	94.32	5.04	<0.001	<0.001	0.011	0.817	
12–13	63	95.46	4.50	<0.001	<0.001	< 0.001	0.248	0.903

#### Predictive value of age on performance in the non-word repetition test

To explore the effects of age on NWR performance, simple linear regression was performed to test if participants’ age in months significantly predicted performance in NWR test (in number of syllables). The overall regression was statistically significant [*R*^2^ = 0.198, *F*(1, 385) = 96.03, *p* < 0.001]. It was found that participants’ age (*B* = 0.164, *p* < 0.001) significantly predicted the performance in NWR task which means that children gain approximately 2 raw points (i.e., 2 syllables) per year, at least at the age range that we examined.

### Normative data for the NRW task

Raw scores (number of syllables correctly repeated) obtained by the participants aged 7–13 were transformed into percentiles. Table 4 presents normative data for the NWR test for each age range and indicates which is the expected average (50th percentile), below average, and above average performance for each range. For example, it indicates that the expected average score for children aged 7–8 years old is 93 out of 108 syllables, which is equal to the 50th percentile, i.e., 50% of children at this age perform at this level.

**TABLE 4:** Normative data from raw scores (number of correct syllables repeated) for the NWR test in percentiles.

	Age range					

Percentiles%	7–8 (*N* = 54)	8–9 (*N* = 64)	9–10 (*N* = 70)	10–11 (*N* = 61)	11–12 (*N* = 75)	12–13 (*N* = 63)
5	82	78	83	91	93	93
15	86	86	89	95	97	99
25	88	90	93	96	100	101
35	90	93	96	98	100	103
50	93	98	101	102	103	105
65	95	101	103	104	105	106
75	99	103	104	105	106	107
85	102	105	106	106	107	108
95	105	107	108	108	108	108

## Discussion

The present study aimed at filling the gap in the lack of standardized phonological STM assessment tools for Greek-speaking school-aged children (7–13 years old) by validating a test of NWR. Its main goal was to explore NWR skills of typically developing (TD) children in Greek and contribute to the diagnostic value of NWR tasks when used with clinical populations. More specifically, this study explores the validity and reliability of an NWR task in a large cohort of Greek-speaking children attending elementary and secondary school (7–13 years old), divided into six age groups. Our research questions were: (i) whether NWR is a reliable measure for TD Greek-speaking children aged 7–13 years old, (ii) whether NWR test is a valid measure for TD Greek-speaking children, and more specifically, what is the relationship between NWR and reading fluency skills in TD children of this age range and whether performance in NWR can predict reading fluency skills and (iii) what is the relationship between age and performance in NWR test and, more specifically, whether there is an escalation in performance as a function of age group, and whether age can predict performance in NWR test.

In order to investigate the external and internal reliability of the NWR test, a test–retest reliability analysis and a Cronbach’s alpha coefficient analysis were performed, respectively. The results showed an excellent test–retest reliability and a good internal reliability, suggesting that it is a reliable measure for TD Greek-speaking children aged between 7 and 13 years.

In order to investigate the validity of the NWR test, correlation analysis was conducted and significant correlations were found between NWR and reading fluency for the whole group and for four of the six age groups (for ages 9–10 and 12–13) for the group 9–10, which was not significant but weak and for the group 12–13 years, which was not significant and negligible. These results suggest that the association between non-word repetition and reading ability is as strong for Greek language as it is for English (Maridaki-Kassotaki, 2002) and that the relationship between phonological STM and reading is reciprocal (Gathercole et al., 1992; Gathercole, 1995; Nation and Hulme, 2011), at least up to 12–year-old TD children. Moreover, these findings are in line with studies with children and adolescents with reading and language difficulties as well (Snowling et al., 2000; Catts et al., 2005; Conti-Ramsden and Durkin, 2007; Ebbels et al., 2012). However, regarding, our older group of adolescents (12–13 years old) who are skilled readers, our findings are not in line with the above-mentioned studies from reading and/or language-impaired children, maybe because for our TD adolescents reading fluency skills have become automated and are no longer affected by NWR skills and vice-versa, contrary to what applies to younger TD children and to reading and/or language impaired children and adolescents. To the best of our knowledge, there is no study with TD adolescents over 12 years old that has demonstrated a significant correlation between NWR skills and reading fluency. As far as the 9–10-year-old group is concerned, the correlation between NWR and reading fluency skills might not have been significant but it was weak, suggesting that at this age range and for TD children NWR skills no longer significantly but weakly affect reading skills, since as mentioned earlier in our results, performance start to reach a ceiling.

In order to strengthen the internal validity of the NWR test, we performed regression analysis to explore whether performance in NWR predicts reading fluency skills. We found that performance in NWR contributed significantly to performance in reading fluency, corroborating the results of other studies with TD and reading impaired children (e.g., Conti-Ramsden and Durkin, 2007; Rispens and Baker, 2012; Schuchardt et al., 2013; Fischbach et al., 2014) suggesting that the NWR test can screen for children with or without reading impairment, because NWR skills reflect phonological processing skills, which are indispensable for reading acquisition (Snowling et al., 1991; Bowey, 2001; Nation and Hulme, 2011). Consequently, inadequate phonological processing skills lead to reading acquisition problems. Moreover, NWR skills are a reflection of children’s phonological representations, which are involved in language learning and play a causal role in language development (Baddeley et al., 1998; Snowling, 2006; Coady and Evans, 2008). This is why NWR task is considered to be a marker of DLD (see Schwob et al., 2021, for meta-analysis). These results confirm the fact that the NWR task is valid to screen not only for TD but also clinical populations, such as children with reading impairment (Melby-Lervåg and Lervåg, 2012; Ehrhorn et al., 2021) or DLD (Graf Estes et al., 2007; Schwob et al., 2021).

In order to investigate the relationship between age and performance in the NWR test, we first examined whether the relevant scores increase as a function of age group. One-way Anova tests revealed significant effects for NWR. *Post-hoc* tests revealed significant differences between groups that differed in 2 years or more and this difference was no longer significant after 10 years of age. This finding suggests that phonological STM increases in capacity along with age, but only until the age of 10, where it seems to reach a ceiling. This finding is partly in line with Gathercole (1998), who claims that memory function shows a gradual improvement from childhood to early adolescence. The current results are also consistent with previous studies providing normative data from NWR tasks, showing a gradual and steady increase in performance as children grow older (Baddeley and Gathercole, 1996; Pickering and Gathercole, 2001; Simkin and Conti-Ramsden, 2001) and they show that the NWR ability follows a developmental pattern in Greek-speaking school-aged children and adolescents, with young children having more difficulty repeating 3–6 syllable non-words than older children. However, our findings are not in line with studies that show that adolescents of 14–15 years reach adult-like levels (Gathercole and Alloway, 2006; Gathercole et al., 2006), since the children in our study reached a ceiling earlier at 10 years and not at adolescence. Our results also add to the existing body of research that shows that the involvement of phonological STM may vary at different ages and different levels of language competence (Marecka et al., 2018).

In addition, it was examined whether the participants’ chronological age could predict performance in NRW task. Linear regression analysis showed that the participants’ age contributed significantly to performance on NWR, suggesting that performance in NWR can discriminate 7–13-year-old children and adolescents in different age groups.

Among the limitations of our study is that the sample size is considerable for validating the NWR task, but not large enough for standardizing the task. Therefore, the results should not be generalized and should be interpreted with caution. A larger sample that is representative of the general population from around Greece would contribute to the generalization of the results. Moreover, additional studies with clinical populations (e.g., children with reading or language impairment) would qualify the use of the test as a screening tool in the clinical practice.

Our study contributes to the research by providing normative data from an NWR task in the Greek language for school-aged children 7–13 years old and by bridging the gap in the Greek language with norms for children aged 9–13 years old. It can be concluded, thus, that this NWR task can be successfully used as a reliable and valid measure of phonological STM at least in the age range that was examined in this study. Finally, our findings have additional clinical implications, since the NWR task can be used not only in the typical population but also as a screening tool for clinical populations, such as children with language or reading disorders.

## Data availability statement

The raw data supporting the conclusions of this article will be made available by the authors, without undue reservation.

## Ethics statement

Ethical review and approval was not required for the study on human participants in accordance with the local legislation and institutional requirements. Written informed consent to participate in this study was provided by the participants’ legal guardian/next of kin.

## Author contributions

All authors contributed to the conception and design of the study, interpretation of data, drafting or revising of the article, and approved the final version of the manuscript.
